# Dynamic management of traumatic brain injury in rat: injectable hydrogels and future directions

**DOI:** 10.3389/fneur.2026.1824220

**Published:** 2026-04-30

**Authors:** Hongyan Jiang, Xinyuan Luo, Hengxi Li, Zhiying Guo, Yan Cao, Li Yang, Haiying Wu, Ping Li

**Affiliations:** 1 Department of Anatomy and Histology and Embryology, Faculty of Basic Medical Science, Kunming Medical University, Kunming, Yunnan, China; 2 Department of Emergency and Intensive Care Unit, The First Affiliated Hospital of Kunming Medical University, Kunming, China

**Keywords:** animal model, biomaterial, experimental brain injury, injectable hydrogel, rat brain injury

## Abstract

The precise treatment and dynamic management of traumatic brain injury pose a substantial challenge to the global healthcare sector. This challenge arises not only from the complex and dynamically evolving pathological microenvironment that follows brain injury and the heterogeneity of the damage but also from the critical importance of maintaining the structural integrity of brain tissue. The intricate pathophysiological milieu subsequent to traumatic brain injury (TBI) hinders the reparative processes, thereby rendering current therapeutic strategies insufficient to satisfy clinical treatment demands. Injectable hydrogels represent a novel and promising biomaterial, offering a groundbreaking platform technology for the repair of traumatic brain injuries. Their distinctive benefits, such as minimally invasive delivery, *in situ* molding, self-healing capabilities, and adaptability to irregular injury sites, position them as an emerging approach in this field. This study provides a systematic review of the current research landscape and pathogenesis associated with TBI, followed by an in-depth analysis of the application of injectable hydrogels in TBI management. It further explores the use of injectable hydrogels across various TBI models and concludes with a discussion on prospective developmental trajectories. The objective is to elucidate the significance of injectable hydrogels in addressing the limitations of conventional therapies, thereby advancing the implementation of innovative therapeutic strategies for TBI treatment and repair.

## 1 Introduction

Traumatic brain injury (TBI) is characterized by its acute onset, rapid progression, high rates of disability and mortality, and poses a serious threat to human life and health (1, 2). The pathological progression of TBI is predominantly characterized by primary and secondary injuries. Primary injury refers to the immediate mechanical damage to cerebral tissue, which may include skull fractures, intracranial hemorrhage, cerebral contusion, and axonal injury. If primary injury is not addressed with timely and effective intervention, secondary injury ensues within minutes of the initial trauma and can persist for years. This secondary phase is mainly associated with disruptions in ionic homeostasis, excitotoxicity, oxidative stress, inflammation, and cell death (3). In comparison to primary brain injury, secondary brain injury causes far more severe damage, often leading to more serious neurological dysfunction and lasting functional impairment, ultimately resulting in a significant deterioration in the patient’s quality of life (4).

Considering the intricate pathogenesis and pathological features of TBI, achieving effective clinical treatment continues to be a formidable challenge. Nonetheless, the advancement of pharmacological treatment strategies for TBI is confronted with several challenges. Primarily, the delivery of drugs to the brain is often hindered by the blood–brain barrier (BBB), which can obstruct the passage of therapeutic agents to the site of injury, thereby limiting their efficacy (5, 6). Furthermore, the distinctive pathophysiological attributes of the brain hinder the diffusion, distribution, and retention of drugs, thereby complicating the accumulation of therapeutically effective concentrations within cerebral tissue (7, 8). Study has noted that traditional pharmaceuticals frequently accumulate in various organs, which can lead to systemic adverse reactions (7). It is precisely because of the limitations in using traditional drugs for TBI that biomaterials have gained prominence.

Given these considerations, there is a pressing necessity to investigate and develop innovative therapeutic strategies aimed at mitigating secondary injury, which could enhance neural network reorganization, and promote functional recovery. The swift advancements in tissue engineering present promising opportunities for brain injury treatment, particularly in the development of biomaterials, offers novel therapeutic approaches and strategies (9). Among the biomaterials, hydrogels are widely utilized for the delivery of diverse therapeutic agents due to their high-water content, biodegradability, and resemblance to natural extracellular matrices. Hydrogels, with their three-dimensional water-soluble network structure, have the ability to replicate the microenvironment of brain tissue (10). In addition, by integrating minimally invasive characteristics with efficient defect-filling capabilities, injectable hydrogels represent promising candidate biomaterials for brain tissue engineering (10), thereby playing a significant role in the management of TBI.

**Figure 1:**
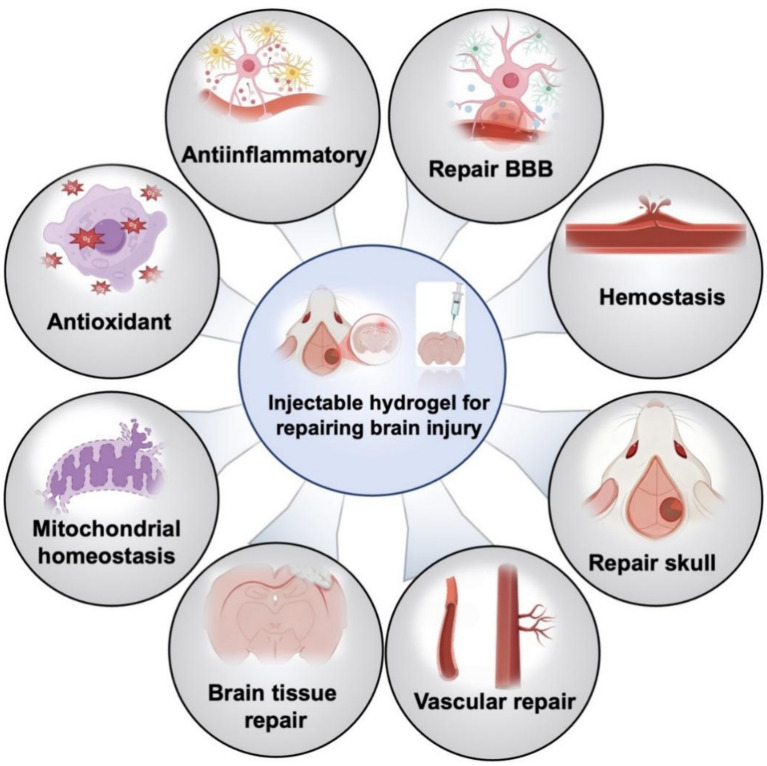
Repair function of injectable hydrogels after traumatic brain injury.

This study provides a comprehensive review of the current research landscape concerning the use of injectable hydrogels in the treatment of TBI, with the objective of elucidating potential therapeutic strategies for this condition. Initially, the study delineates the existing research framework and the pathogenesis associated with TBI. It then conducts a detailed analysis of the application of injectable hydrogels in TBI treatment, highlighting their multifaceted roles in haemostasis, analgesia, anti-inflammatory and antioxidant effects, promotion of angiogenesis, regulation of mitochondrial homeostasis, and facilitation of tissue repair, as depicted in Scheme 1 (see Figure 1). Additionally, the paper examines the utilization of injectable hydrogels across various TBI models.

## 2 Traumatic brain injury

### 2.1 Epidemiological characteristics and current research status of traumatic brain injury

TBI is a neurological disorder precipitated by external mechanical forces that compromise the structure and function of the brain. With approximately 69 million new cases of TBI reported each year all over the world (11), these figures underscore the significance of TBI as a major public health issue and a serious global medical challenge. In America, nearly 1.7 million individuals suffered from traumatic brain injuries annually (12). In China, data from 2019 reveal that the growth rate of patients after TBI increased by 119.4% and the incidence rate among males is double that of females, with a notable rise observed in individuals aged 50 and above, primarily due to traffic accidents (13). In economically developed regions, falls among children and the elderly have surpassed road traffic accidents as the primary cause of TBI (14). An analysis of the epidemiological characteristics of TBI reveals significant variations across countries, highlighting disparities between developed and developing nations, as well as between economically advanced and underdeveloped regions within developing countries. These findings are consistent with the results of a study published in 2025, which noted significant variations in the incidence of traumatic brain injury across countries. The study noted implementing strategies such as reducing alcohol consumption, preventing falls, and strictly enforcing traffic regulations can contribute to a decrease in TBI occurrences (15). Additionally, improving the efficiency of medical treatments and enhancing healthcare and nursing standards positively impact the prognosis for TBI patients (15). In conclusion, the prevention of TBI is crucial in minimizing its occurrence. In the event that an individual sustains such an injury, it is imperative to administer prompt, early, and effective treatment, accompanied by continuous monitoring of the patient’s prognosis.

### 2.2 Pathogenesis of traumatic brain injury

**Figure 2:**
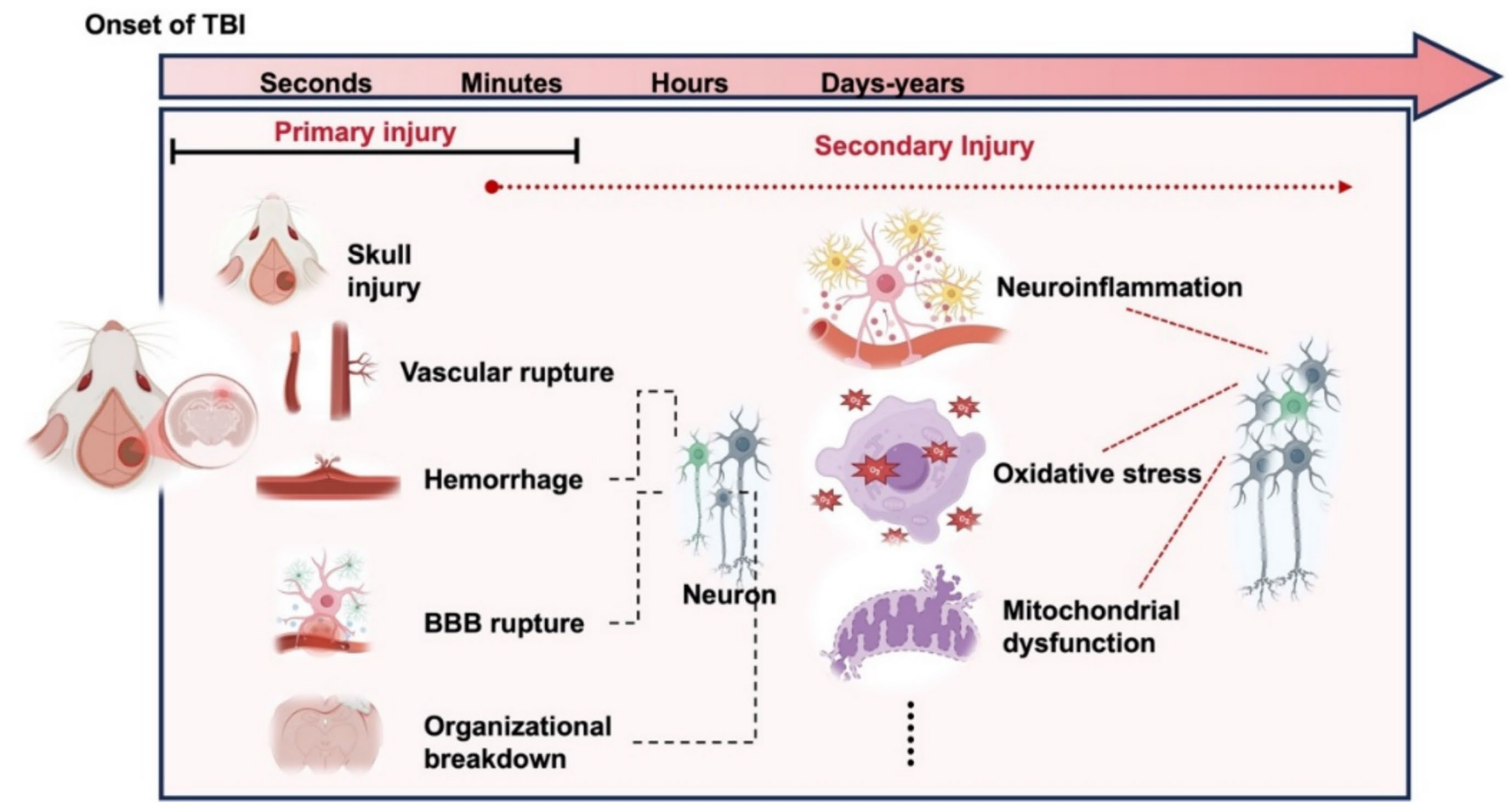
Primary and secondary injuries caused by traumatic brain injury.

As illustrated in Figure 2, the pathophysiological process associated with TBI predominantly involves a primary injury that is directly induced by mechanical impact. And then followed by molecular and cellular-level disturbances leading to secondary pathological damage. This includes excitotoxicity, oxidative stress, mitochondrial dysfunction, ionic imbalances, and neuroinflammation. The release of glutamate, reactive oxygen species(ROS), and inflammatory cytokines resulting from these pathological changes further induces apoptosis, thereby exacerbating brain injury (16). Subsequently, we provide a comprehensive summary and delineation of the pathological injury mechanisms associated with TBI.

#### 2.2.1 Relationship between neuronal Excitotoxicity and traumatic brain injury

After TBI, there is a significant disturbance in ionic homeostasis, such as sodium, potassium, calcium, and chloride, initiating a cascade of secondary injuries that ultimately lead to persistent neurological dysfunction (17). This ionic dysfunction persists after TBI and substantially influences the prognosis of affected patients (18). Primary injury caused by TBI directly disrupts the cellular membrane integrity of neurons, axons, glial cells, and blood vessels (19). This leads to the instantaneous loss of the normal resting membrane potential in neuronal cells, the massive release of excitatory amino acids, and the abnormal opening of voltage-gated ion channels. Consequently, large influxes of extracellular Na^+^ and Ca^2+^ flood into the cell (19, 20), which could directly mediate excitotoxicity. TBI disrupts the BBB, leading to excessive neurotransmitter release. The over-release of glutamate causes hyperactivation of ionotropic glutamate receptors (NMDA and AMPA), facilitating the influx of extracellular Na^+^ and Ca^2+^ ions into cells. This disrupts ionic homeostasis, with excessive Ca^2+^ activating apoptosis-inducing proteins, ultimately resulting in cell death (19, 21, 22). Research has confirmed that glutamate-mediated excitotoxicity acts as a key role in the acute phase of secondary injury following TBI (23). Following brain injury, glutamate is released into the synaptic cleft, binding to N-methyl-D-aspartate (NMDA) and *α*-amino-3-hydroxy-5-methyl-4-isoxazolepropionic acid (AMPA) receptors. This leads to increased Ca^2+^ influx, thereby exacerbating excitotoxicity cascades (24). A 2022 study demonstrated that low molecular weight dextran sulphate restores glutamate homeostasis and amino acid metabolism following severe TBI, significantly improving mitochondrial function in rats with severe TBI (25). Research has demonstrated that high concentrations of glutamate (100–500 μM) can damage neurons, inducing cell death *in vitro* (26, 27). Following ischemia–reperfusion in rats, administration of NMDA receptor antagonists can reduce cell death and enhance neuroprotection (28). However, clinical trials have revealed that NMDA antagonists do not produce significant neuroprotective effects in injury models (29, 30). Consequently, the application of NMDA antagonists in TBI requires further investigation (31).

Based on the aforementioned research, we have acquired new insights into glutamate-mediated excitotoxicity as an important mechanism underlying secondary injury following TBI. Modulating glutamate homeostasis and inhibiting its toxic pathways may offer promising therapeutic strategies. In summary, the disruption of ionic homeostasis induced by TBI represents a dynamic, complex, and systemically impactful pathological process. This disruption acts both as a direct cause of energy crises, edema, excitotoxicity, and oxidative stress, and as a consequence of the exacerbation of these processes. Integrated therapeutic approaches targeting various stages of ionic dysregulation hold potential for advancing the prognosis of TBI.

#### 2.2.2 Relationship between oxidative stress damage and traumatic brain injury

Brain is a highly oxygen-dependent organ, which use approximately 20% of the body’s total oxygen supply. In the aftermath of brain injury, cerebral oxygen consumption increases, thereby elevating the potential for the production of ROS (32). TBI induces excitotoxicity while concurrently initiating oxidative stress, which is centered on the excessive generation of ROS and represents a critical pathogenic factor in the cascade of secondary injuries. Oxidative stress disrupts the balance between ROS production and clearance. Excessive nitric oxide (NO) reacts with free radical peroxides to form peroxynitrite, further exacerbating oxidative damage. The persistent release of ROS and subsequent lipid peroxidation negatively impacts cerebral blood flow, thereby impeding neural plasticity following TBI (17). Extensive research demonstrates that oxidative stress plays a significant role in the pathogenesis of cerebral edema across various models of animals after TBI. This pathological process is characterized by mitochondrial dysfunction, disruption of the BBB, sensorimotor deficits, and subsequent neuronal damage, ultimately exacerbating the severity of brain injury (33–38).

In conclusion, oxidative stress serves as a critical role in the sequence of secondary damage processes. Its sustained presence results in the accumulation of Ca^2+^ ions, which subsequently activates nitric oxide synthase (NOS) to produce excessive amounts of NO. This NO then reacts with superoxide to form highly reactive oxidants, thereby perpetuating oxidative damage and lipid peroxidation. As a result, oxidative stress adversely impacts cerebral blood flow, immune function, and neural plasticity.

#### 2.2.3 Relationship between mitochondrial damage and traumatic brain injury

Mitochondrial structural and functional abnormalities represent a critical pathological factor in neuronal death. As the cellular powerhouse, mitochondria are essential for sustaining energy supply, regulating apoptosis, and maintaining the equilibrium between oxidative stress and calcium homeostasis. Upon impairment of mitochondrial function, a cascade of events is triggered, including energy depletion, increased oxidative stress damage, activation of apoptotic pathways, and disruption of calcium homeostasis, which generally culminate in neuronal death (39). As it reported, TBI leads to overt structural abnormalities in mitochondria, including swelling, vacuolation, and disruption or loss of cristae—folds of the inner mitochondrial membrane. These morphological alterations are intimately linked with oxidative stress, calcium dysregulation, energy deficits, inflammatory responses, and cell death. Collectively, these processes influence the clinical severity and long-term prognosis of TBI (40). Therefore, a comprehensive investigation into mitochondrial alterations following brain trauma provides valuable insights for the treatment and prognosis of TBI.

A study has observed that mitochondria in distinct neuronal subtypes display differential sensitivity to injury. Notably, mitochondrial dysfunction in microglia induces their polarization towards a pro-inflammatory phenotype, thereby intensifying neuroinflammation (41). Endothelial cell mitochondrial dysfunction is the key factor in BBB disruption (42). Studies have identified nuanced gender differences in mitochondrial dysfunction following TBI. Specifically, non-synaptic mitochondria in female animals exhibit earlier bioenergetic impairment, while male animals show earlier damage in synaptic mitochondria. These findings highlight the critical importance of incorporating gender as a biological variable in research (43).

In conclusion, it is imperative to acknowledge that mitochondrial dysfunction following brain injury does not manifest uniformly across all cellular types, nor is it restricted to a singular neuronal cell type. Further study on mitochondrial damage in neuronal cells post-brain injury should focus on identifying specific cell types that exhibit significant mitochondrial impairment, refining subcellular classifications, and, where possible, conducting studies on brain trauma. Furthermore, investigating the variations in mitochondrial damage in neuronal cells across genders presents new research opportunities. A comprehensive understanding of the intricate mechanisms underlying mitochondrial damage after TBI not only elucidates fundamental principles governing secondary damage in such injuries but also establishes a solid theoretical basis and innovative pathways for the development of targeted mitochondrial neuroprotective therapies.

#### 2.2.4 Relationship between neuroinflammation and traumatic brain injury

TBI does not constitute a singular mechanical event; rather, the ensuing pathophysiological processes, notably the neuroinflammatory cascade, are critical contributors to secondary brain damage, neurological deficits, and potentially long-term neurodegeneration (44). The neuroinflammatory cascade following TBI directly compromises the integrity of the BBB, leading to the infiltration of immune cells and plasma proteins from the bloodstream into the brain parenchyma (45, 46). Activated immune cells and damaged neurons secrete significant amounts of inflammatory mediators, such as Interleukin-1 beta (IL-1β), Interleukin-6 (IL-6), Tumor Necrosis Factor-alpha (TNF-*α*), chemokines, ROS, among others, thereby establishing a self-amplifying inflammatory signaling network. The escalation and persistence of this inflammatory cascade are contingent upon the activation of multiple intracellular signaling pathways and intricate intercellular communication among various cell types. Recent study has further demonstrated that following BBB disruption, fibrinogen accumulates at the blood vessel-astrocyte interface, where it directly activates astrocytes and thereby promotes neuroinflammatory processes (47). However, neuroinflammation following TBI is not entirely detrimental, primarily serving a protective function during the acute phase: in the initial stages of injury, the inflammatory response aids in isolating the damaged area, clearing cellular debris, and initiating tissue repair processes (48). The inflammatory response in the chronic phase is primarily characterized by destructive effects (49).

In conclusion, TBI initiates and intensifies neuroinflammatory cascades by compromising the integrity of the BBB, activating innate immune cells, and triggering intricate intracellular signaling pathways and intercellular communication. This process is characterized by spatiotemporal dynamics and duality: although inflammation may be beneficial in the initial phase, it becomes a central pathological mechanism during recovery, contributing to secondary brain injury, neurodegeneration, and systemic multi-organ complications. This understanding has significant implications for clinical therapeutic strategies. Indiscriminate systemic anti-inflammatory treatments have not consistently demonstrated efficacy in TBI and may even be harmful. Future therapeutic approaches should prioritize precise modulation of inflammation rather than its blanket suppression. Additionally, managing systemic inflammation following TBI is crucial for improving patient prognosis.

### 2.3 Challenges and opportunities in the treatment of traumatic brain injury

With the rapid advancement of medical technology, research into the management of TBI has proliferated, leading to improved patient outcomes. Pharmacological treatment for TBI has shifted from symptom-specific control towards a pathophysiology-driven, full-cycle management approach: the acute phase centers on neuroprotective agents and the prevention and treatment of complications (50). It is heartening to note that natural products have demonstrated certain potential in the treatment of TBI. The following Table 1 presents the star compounds currently under investigation.

**Table 1:** Star natural drugs in TBI treatment.

Natural product	Pharmacological effects	Adverse effects
Cannabidiol	can pass through the BBB (137); Inhibit excitotoxicity (138, 139); Anti-inflammatory, anti-apoptotic, improvement in BBB integrity, weight gain (2, 3, 140); Alleviate anxiety and insomnia, relieve pain, stimulate appetite, and combat depression. (141, 142); Anti-inflammatory, prolonging lifespan (143); Synaptic remodelling (131); Repair gastrointestinal damage and promote bone healing (144).	Low oral bioavailability, first-pass effect, gastrointestinal reactions; Animals: Among animals, CBD adverse events include developmental toxicity, embryo-foetal mortality, central nervous system depression and neurotoxicity, hepatocyte damage, reduced spermatogenesis, alterations in organ weight, changes in the male reproductive system, and hypotension (145); Human: Liver abnormalities, diarrhoea, fatigue, vomiting and drowsiness (145).
Berberine	Inhibits excitotoxicity and exhibits anti-inflammatory properties (102, 146–149); Antioxidant (146, 148, 149); Anti-anxiety, anti-depressant (147); Anti-apoptotic, improving morphine tolerance (149).	Hydrophobic drugs, low bioavailability (150)
Curcumin	Reduce oxidative stress and inflammation (104, 151–161); Maintain and promote microbial balance (155); Promote angiogenesis (156); Neurogenesis and Cognitive Recovery (157, 162); Pain relief (163); Regulate the synthesis, biotransformation and transport of thyroid hormones (160).	Low oral bioavailability, gastrointestinal reactions (164)
Melatonin	Regulating endoplasmic reticulum stress (165), Inhibition of ferroptosis (166), Anti-inflammatory, reduces cerebral oedema, and exerts neuroprotective effects (167); antidepressant (168); Antioxidant damage and mitochondrial dysfunction (107).	Headache, dizziness, drowsiness (169).

Although existing research has reported natural compounds for the treatment of TBI, these therapies are frequently constrained by the BBB, resulting in poor efficacy following oral or intravenous administration, low effective concentrations, and the presence of off-target effects (51, 52). Concurrently, the transition of natural product applications from laboratory to clinical settings necessitates large-scale, standardized clinical trials, indicating that the prospects for translational research warrant additional validation.

## 3 Hydrogel

### 3.1 Definition and classification of hydrogels

Hydrogels are a class of macromolecules containing hydrophilic groups, forming polymeric systems through covalent bonds, hydrogen bonds, and van der Waals forces. These polymeric materials possess a porous internal structure capable of absorbing substantial quantities of water and supporting high-load compounds. They absorb large numbers of water molecules and swell, yet do not dissolve (53). Due to their remarkable water solubility, biocompatibility, extensibility, and adaptability, hydrogels have become prominent materials in biomedical therapies (54, 55), holding an indispensable role in the fields of precision medicine and regenerative medicine.

Hydrogels may be categorized by chemical composition into natural and synthetic hydrogels. Natural hydrogels primarily utilize natural polymers as their matrix, chiefly comprising polysaccharides or peptides such as sodium alginate, chitosan, collagen, sericin, and agarose. These exhibit high biocompatibility, degradability, but their mechanical strength is generally low (56, 57). Hydrogel materials derived from natural polysaccharides exhibit high water retention, self-healing, biodegradability, biocompatibility, and non-toxicity, rendering them suitable as medical materials for in-vivo implantation. Synthetic hydrogels, formed through covalent or physical cross-linking of artificially synthesized polymers, offer advantages such as controllable structure, high mechanical strength, and excellent stability. However, their biocompatibility and degradability often require optimization. Key examples include polyacrylic acid, polyethylene glycol, polyvinyl alcohol, and polyacrylic acid, whose stability can be chemically regulated (57).

Natural hydrogels boast biocompatibility as their core advantage, while synthetic hydrogels excel in their performance designability (58). To address the limitations inherent in natural hydrogels, such as inadequate mechanical strength, restricted functionality, uncontrolled degradation rates, and the suboptimal biocompatibility associated with synthetic hydrogels, future research should prioritize comprehensive optimization through biomimetic modification. Future advancements will focus on more accurately replicating the complex physiological microenvironments of specific tissues, while ensuring the safety and efficacy necessary for clinical translation.

Injectable hydrogels have attracted widespread interest in biomedicine due to their unique rheological properties and tunable performance. Their rheological behavior is characterized by a high viscosity at low shear rates, ensuring proper flowability during injection, and a low yield stress, which allows a rapid sol-to-solid transition after injection to secure *in vivo* stability (59, 60). Injectable hydrogels serve as an ideal drug delivery system, as they not only release medication in a controlled manner and prolong its action, but also accumulate within brain on the injured site. This simultaneously enhances therapeutic efficacy while minimizing adverse effects on healthy tissues (61–64), which also serves as a reminder that during the treatment of TBI, drug delivery can be achieved through the preparation of injectable hydrogels, enabling sustained therapeutic effects.

### 3.2 Applications of injectable hydrogels

Injectable hydrogels showed a significant therapeutic efficacy in clinical disease management owing to their flexibility, biocompatibility, and ease of modification. Research into myocardial injury has revealed that these hydrogels can be administered via endocardial, epicardial, or coronary artery injection without requiring invasive surgery, thereby substantially reducing surgical trauma to the body (65). The same report documented research on injectable hydrogels in cases of uncontrollable bleeding caused by acute trauma, confirming the superior haemostatic efficacy of injectable hydrogels (66). The recent study has suggested that the administration of imidazole-based hydrogels significantly promotes tissue repair, consequently mitigating the formation of cystic cavities following spinal cord injury in rat models (67). In a rat model of hemorrhagic brain injury, injection of an extracellular matrix hydrogel into the lesion cavity effectively promoted neuronal migration and tissue regeneration (68). In rats with spinal cord injury, injection of a hyaluronic acid hydrogel promoted angiogenesis, neuronal regeneration, and subsequent spinal cord repair (69).

Based on the aforementioned findings, we contend that injectable hydrogels hold significant promise in the treatment of both acute and chronic diseases. By tailoring material design to the pathological alterations of specific conditions, these hydrogels can play a crucial role across diverse therapeutic needs.

## 4 The application of injectable hydrogels in traumatic brain injury

Injectable hydrogels exhibit significant potential for therapeutic application in the treatment of TBI. By adjusting the mechanical properties of hydrogels to correspond with the modulus of brain tissue, these materials can more effectively mimic the biomechanical characteristics of soft tissue (70). Due to its fragility, softness, and high elasticity, brain tissue is considered the most delicate tissue within the human body (71, 72). Hence, biomaterials intended for cerebral applications must exhibit several critical properties: superior biocompatibility (72), suitable mechanical characteristics (72, 73), minimal swelling, fluidity, self-healing ability, and degradability (72, 74, 75). Accordingly, the following content will predominantly focus on summarizing and synthesizing the utilization of injectable hydrogels in the management of brain injuries.

### 4.1 Injectable hydrogels for acute haemostasis following traumatic brain injury

The primary concern in TBI is the damage to cerebral tissue and it can lead to the rupture of cerebral vasculature, with the initial several hours post-trauma being particularly critical. If hemorrhaging is not effectively managed, it can lead to severe cerebral edema, which may worsen the condition and potentially result in fatal hemorrhage (76–79). Therefore, rapid haemostasis has become crucial in pre-hospital care and emergency treatment.

Facilitates injectable self-healing through dynamic boronate ester bonds and adheres to brain tissue via catechol interactions, while phenylboronic acid grafted hyaluronic acid/dopamine grafted gelatin hydrogel (HA-PBA/Gel-Dopa) was administered into lesion cavities caused by TBI of mice and it effectively achieve immediate hemostasis during trauma, thereby promoting swift wound closure (80). Similarly, post-traumatic hemostasis presents a significant challenge in the field of research. Existing surgical hemostatic techniques often result in secondary tissue damage and functional impairments. Recent studies have indicated that the administration of Ca^2+^-Crosslinked Oxidized Sodium Alginate (OSA) and Carboxymethyl Chitosan hydrogel (COCS) subsequent to brain injury facilitates haemostasis in the liver and abdominal aorta, thereby expediting the repair of the compromised abdominal aorta (81). The COCS hydrogel establishes a dual-network dynamic crosslinking system through Schiff base interactions and Ca^2+^ coordination, which imparts superior injectability, self-healing ability, and strong mechanical properties (81). These findings offer a foundation for employing haemostatic strategies in the management of TBI (82–84).

Injectable hydrogels encounter numerous challenges in the treatment of haemostasis following TBI. These challenges primarily arise from the discordance between the material properties of hydrogels and the brain’s unique physiological environment, the urgent nature of haemostasis requirements, and the complexity of clinical procedures (80). Critical concerns encompass immune and inflammatory responses, limited degradability, the necessity for stringent precision in injection procedures, and the potential for hydrogel injection to result in space-occupying effects that elevate intracranial pressure, consequently exacerbating brain injury. Nonetheless, these challenges are accompanied by opportunities, highlighting the need for enhanced collaboration and knowledge exchange among the fields of materials science, neurosurgery, and translational medicine in the design of injectable hydrogels for post-traumatic haemostasis. Only through multidisciplinary cooperation to optimize material design, validation systems, and delivery techniques can the true clinical potential of these hydrogels be realized.

Recent study demonstrates that sodium aescinate, which self-assemble into powders from *in situ* sprayed herbal extracts, rapidly form hydrogels upon interaction with liquids (1). This process exerts swift hemostatic effects during the acute phase of TBI. As both mangiferin and rutin possess anti-inflammatory, antioxidant and anti-apoptotic properties, and both form self-assembling gels upon contact with liquids, they can work synergistically to achieve the dual functions of rapid haemostasis and *in situ* neuroprotection required for emergency treatment during the acute phase of traumatic brain injury (85). Specifically, it mitigates severe cerebral edema and inflammatory responses following TBI when applied via in situ spraying, all while avoiding notable toxic side effects (85).

The aforementioned study presents a promising strategy for addressing acute haemorrhagic phases following TBI through the use of natural products. By refining the materials and optimizing both the methods and routes of administration, the approach effectively reduces tissue compression, thereby minimizing secondary damage to the injured brain tissue. Additionally, the use of relatively convenient administration methods further enhances the haemostatic therapeutic efficacy.

### 4.2 Injectable hydrogel alleviates chronic pain following traumatic brain injury

Secondary pain resulting from TBI continues to pose a significant challenge for patients, their families, and healthcare institutions. In America, military personnel who have suffered TBI and experience persistent photophobia, headaches and migraines (86). More than 60% of individuals with TBI experience chronic post-traumatic headaches (87). Consequently, pain management during the recovery phase for patients with TBI warrants particular attention (88). Traditional postoperative pain management has primarily relied on intravenous administration of opioid medications (89). However, the risk of serious adverse reactions and addiction limits its application (90). To mitigate these limitations, a recent study incorporated Rhodojaponin III (Rhodojaponin III is a gray-alkaloid-type diterpene isolated from medicinal plants, which possesses anti-inflammatory and analgesic properties) into a colloidal drug delivery system comprising hydroxypropyl trimethylammonium chloride-modified solid lipid nanoparticles (HACC-SLNs) for oral administration, which enhanced the pharmacokinetic profile of Rhodojaponin III, prolonged its duration of action, and demonstrated beneficial effects in the treatment of chronic pain (89).

Research indicates that hydrogels infused with ropivacaine extend the duration of relief from chemotherapy-induced peripheral neuropathic pain, thereby affirming their beneficial analgesic properties (91). In a rat model of sciatic nerve block, bupivacaine-loaded poloxamer/oxidized hyaluronic acid hydrogel (Bup/PO) reduced inflammatory responses, and facilitated the sustained release of bupivacaine, thereby providing analgesic effects and presenting a promising approach for the long-term management of postoperative pain (92). Similarly, in a mouse model of sciatic nerve compression, hyaluronic acid-poly(vinyl alcohol)-heparin hydrogel (HA-PVA-Hep) were shown to effectively enhance sensory-motor function impaired by peripheral nerve injury, mitigate inflammatory responses, and alleviate pain (93). These studies collectively offer novel insights into the management of chronic pain following brain trauma.

Currently, the clinical management of brain injuries primarily involves the use of non-steroidal anti-inflammatory drugs (NSAIDs) such as aspirin or corticosteroids to attenuate inflammatory responses and alleviate pain, despite their association with significant toxic side effects. Recent research suggests that polydopamine-mediated poly(3,4-ethylenedioxythiophene)-modified cellulose nanowhisker@dGel hydrogel (PPCNW@dGel) have the potential to reduce inflammatory reactions and provide analgesic effects (94, 95). Consequently, the design and development of drug-loaded hydrogels for localized injection are of considerable clinical importance to mitigate the adverse effects associated with prolonged medication use. Localized hydrogel injections facilitate sustained drug release, thereby offering analgesic benefits while minimizing the inconvenience of repeated or long-term medication. This method also enhances the precision of local drug delivery, reducing systemic side effects commonly linked to medication use, and ultimately improving patients’ quality of life. This highlights the necessity of prioritizing pain management for patients with TBI during both the postoperative and recovery phases in contemporary research initiatives.

### 4.3 Injectable hydrogels for sustained anti-inflammatory and antioxidant effects throughout the progression of traumatic brain injury

Following TBI, neuroinflammation occur in nearly all patients. Furthermore, neuroinflammation may exacerbate excessive ROS, thereby exacerbating brain damage (96, 97). Research has shown that hydrogels loaded with carboxymethyl chitosan, oxidized dextran, and dexamethasone (DEX) enhance DEX loading capacity. Following *in situ* injection, they enable sustained local release around lesions, improving neurological function by restoring BBB integrity and suppressing neuroinflammation post-TBI, while effectively avoiding peripheral and central side effects (98). In mice with TBI, in situ injection of cryogenic poloxamer hydrogel loaded with 3-iodothyronamine (Pol/T) reduced local oxygen consumption, maintained BBB integrity, diminished inflammatory responses and cerebral oedema, and promoted functional recovery post-trauma (99). *In situ* injection of a hydrogel delivering triiodothyronine amine (T1AM) following TBI enables localised hypothermia therapy, avoiding the side effects of systemic cooling, mitigating inflammatory responses and cerebral oedema, and promotes functional recovery in mice after TBI (99).

Secondary injury arising from oxidative stress was also constitutes a decisive factor in the prognosis of TBI. A smart hydrogel formed with polyethylene glycol-ROS responsive thioketal linker/2-deoxyglucose hydrogel (P-RT/2DG) into the target region enables precise therapeutic delivery, which confirm that the P-RT/2DG hydrogel reduces oedema, inhibits neuronal apoptosis and neuroinflammation, maintains BBB integrity, and exhibits no systemic toxicity (100). Concurrently, experiments on oxidative stress injury following brain trauma have demonstrated that injection of phenylboronic acid grafted hyaluronic acid/polyvinyl alcohol/deferoxamine hydrogel (HA-PBA/PVA/DFO) reduces oxidative stress, thereby protecting neurons (101). Researchers investigating curcumin’s real-time therapeutic effects in TBI injected curcumin-loaded lysine/poly (ethylene glycol) diacrylate (PEGDA) hydrogels into irregular brain lesion sites. And through magnetic resonance imaging assessed the hydrogel’s real-time therapeutic efficacy, revealing that curcumin-loaded hydrogels suppressed inflammatory responses and promoted neural repair, which offering a promising strategy for injectable hydrogel delivery of natural products in treating TBI (102, 103).

The secondary inflammatory cascade following TBI is closely associated with oxidative stress damage. Studies have reported that triglycerol monostearate/poly (propylene sulfide)120 embedded Curcumin Hydrogel (TM/PC), when injected into the cavity at the injury site in mice, can reduce ROS, decrease neuroinflammation, and promote neuroregeneration and functional recovery (104). When gelatin methacryloyl-poly(propylene sulfide)/procyanidins hydrogel (GelMA-PPS/PC) was applied to the surface of brain tissue directly, which can modulate cellular oxidative stress responses, and synergistically reduces ROS to exert neuroprotective effects (105).

This underscores the necessity for future research to ground the intelligent design of materials in the dynamic pathological changes associated with TBI. Such an approach is anticipated to more accurately mirror the precise and adaptive management of TBI, thereby offering a robust scientific foundation and therapeutic strategies for the treatment and rehabilitation of clinical TBI patients.

### 4.4 Injectable hydrogels for maintaining mitochondrial homeostasis following traumatic brain injury

Mitochondrial energy production is essential for maintaining normal brain function. Following TBI, there is an increasing demand for cerebral energy, which leads to the activation of mitochondrial respiration and an associated rise in ROS production (106). The functionality of mitochondria is significantly impaired, primarily evidenced by a reduced capacity for oxidative phosphorylation, decreased adenosine triphosphate (ATP) production, and elevated ROS levels (107, 108). Additionally, a reduction in mitochondrial membrane potential and the opening of the mitochondrial permeability transition pore are frequently observed, further exacerbating cellular damage (109, 110). Consequently, the prompt assessment of mitochondrial dynamics post-brain injury is crucial for both therapeutic intervention and prognostic evaluation.

The hydrogel is constructed from tannic acid (TA) and quaternized chitosan (QCS). TA endows the system with tissue-adhesive, antioxidant, and chelating capabilities, while QCS contributes cationic charge-mediated antibacterial activity and tissue adhesion. The two components self-assemble into a dynamic, self-healing, and injectable network through hydrogen bonding and electrostatic interactions. Functionally, TA promotes rapid haemostasis and scavenges ROS, whereas QCS prolongs hydrogel retention at the lesion site. Collectively, these mechanisms act synergistically to suppress neuroinflammation and alleviate cerebral oedema (111). In models of hepatocellular carcinoma (112), chronic diabetic injury (113), and myocardial ischaemia-reperfusion (114), it has been confirmed that treatment via injectable hydrogels targeting mitochondria exerts positive antioxidant effects and modulates mitochondrial damage.

### 4.5 Injectable hydrogels for tissue repair following traumatic brain injury

Injectable hydrogels demonstrate synergistic effects through various mechanisms in the treatment of TBI, significantly enhancing bone tissue regeneration and neural repair following cerebral damage. By employing mechanisms such as scaffold formation, controlled release of bioactive factors, immunomodulation, and coordinated vascular-neural regeneration, these hydrogels accomplish dual objectives: the restoration of bone tissue structure and the rehabilitation of neural function in the management of TBI. This approach presents an innovative strategy for clinical translation (70).

#### 4.5.1 Injectable hydrogels for cranial repair following traumatic brain injury

Bone is one of the most complex tissues in the human body. Under appropriate physiological conditions, it possesses the capacity for spontaneous regeneration and healing. However, when severe bone damage occurs due to trauma, its self-repair capabilities become severely limited, resulting in the inability of damaged bone tissue to heal independently (115). Consequently, repairing cranial defects remains a major challenge in neurosurgery. Compounded by poor self-healing capacity, prolonged treatment cycles, and difficult healing, this also imposes a heavy financial burden on patients. There is an urgent need to design composite materials that support bone regeneration while minimizing complications such as infection and inflammation for the treatment of TBI, which may help alleviate this clinical challenge. Among these, injectable hydrogels represent the optimal materials for mimicking the natural environment surrounding human tissues and are considered candidate materials for neurosurgical and cranial reconstruction applications (116).

Upon administration of a Pickering emulsion hydrogel stabilized with nano-hydroxyapatite into a murine cranial defect model, there was a notable bone formation was observed at the defect site, suggesting the system’s potential efficacy in promoting bone repair (117). *In vitro* investigations demonstrated that the polydopamine-gentamicin-alendronate-hydrogel (PDA-G-A-H) intervention in MC3T3-E1 cells significantly enhanced mineralization and osteogenic differentiation, while simultaneously upregulating the expression of osteogenic markers, which suggest its potential applicability in translational research for cranial defect repair and other bone regeneration therapies (118). The incorporation of poly(dl-lactide-co-glycolide)-hyaluronic acid (PLGA-HA) nanoparticles into heparin-modified hyaluronic acid adipic dihydrazide/gelatin methacryloyl hydrogel (HAAC/GelMA), in conjunction with the synergistic effects of IL-10 and ICA significantly enhanced the bone immune microenvironment and facilitated osteogenic differentiation in both *in vitro* and *in vivo* contexts, thereby markedly promoting bone remodeling (119). Study have shown that the administration of 4-aminobenzeneboronic acid grafted oxidized hyaluronic acid/rosmarinic acid/laponite injectable hydrogel (AOHA-RA/Lap) was injected into sites of cranial defects with infections exhibits significant osteogenic properties (120). In a comparable study utilizing a rat cranial defect model, hydrogels incorporating thiolated chitosan-modified paclitaxel liposomes demonstrated efficacy in enhancing osteoblast proliferation and modulating the Wnt signaling pathway, thereby facilitating the repair of cranial defects in rats (121). Likewise, the research has similarly demonstrated that *in vitro* experiments reveal the substantial potential of methylpropenylated hyaluronic acid/chitosan oligosaccharide/mesoporous silica nanoparticles@bone morphogenetic protein-4 hydrogel (HAMA/COS/MSN@BMP-4) in promoting the proliferation and osteogenic differentiation of mouse embryonic osteoblast precursors. And *in vivo* studies have confirmed that this hydrogel effectively fills and seals irregular cranial defect surfaces, significantly enhancing bone regeneration and repair capabilities (122). This suggests an innovative therapeutic strategy for the treatment of bone defects.

Cranial defects pose significant challenges due to the complexity of repair, protracted healing processes, and increased susceptibility to infection, with pharmacological interventions largely proving ineffective (123). The utilization of injectable hydrogels has partially mitigated these challenges. A comprehensive review of the literature over the past 3 years indicates that injectable hydrogels have been extensively tested in rodent models, including rats and mice. Experimental studies involving both cell lines and primary cell cultures have confirmed the osteogenic differentiation-promoting effects of these hydrogels, resulting in favorable therapeutic outcomes. These findings underscore the necessity of actively pursuing the development of biomaterials tailored for cranial repair, with a particular focus on optimizing biocompatibility, adhesion, anti-swelling properties, and antimicrobial efficacy. This holistic approach is anticipated to enhance the efficacy of these materials in facilitating cranial repair, thereby providing viable treatment options for patients with cranial defects (123, 124). Moreover, further investigation is warranted to elucidate the mechanisms through which these biomaterials facilitate cranial regeneration (see Table 2).

**Table 2:** Current research status of injectable hydrogels in bone repair.

Time	Material	Animal/Cell	Model	Function
2025	Hydrogel integrated with nanohydroxyapatite (nHAP)-stabilized Pickering emulsions	*In vivo*: Mice	Skull defect	Potential for anti-tumour effects and promoting bone repair (117)
2025	PDA-G-A-H	*In vitro*: MC3T3-E1 cell line	Bone formation	Anti-inflammatory, osteogenic (118)
2024	PLGA-HA nanoparticles and heparin-modified HAAC/GelMA composite hydrogels	*In vivo*: Rats *In vitro:* Primary BMSCs	Skull defect, bone formation	osteogenic (119)
2024	AOHA-RA/Lap IH	*In vivo:* Rats *In vitro:* Primary BMSCs	Skull defect, bone formation	osteogenic (120)
2024	CSSH	*In vivo:* Rats *In vitro*: MC3T3-E1 cell line	Skull defect, bone formation	Proliferation, differentiation, osteogenesis, bone regeneration (121)
2024	HAMA/COS/MSN@BMP-4	*In vivo*: Mice *In vitro*: MC3T3-E1 cell line	Skull defect, bone formation	Osteogenesis, bone regeneration (122)

#### 4.5.2 Injectable hydrogels for angiogenesis following traumatic brain injury

TBI predominantly results in neuronal cell death within the cerebral cortex. The prompt transplantation of hyaluronic acid hydrogel post-cortical injury facilitates angiogenesis in the affected cortical region, thereby creating an environment conducive to the survival and maturation of newly generated neurons (125). In analogous models of TBI, the *in situ* administration of hyaluronic acid-collagen hydrogels, enriched with exosomes derived from bone marrow-derived mesenchymal stem cells, has been shown to effectively replicate the brain matrix and facilitate sustained exosome release. This methodology promotes angiogenesis and neurogenesis, thereby enhancing neurological recovery post-TBI (126, 127).

## 5 The reparative effects of injectable hydrogels on brain tissue in various traumatic brain injury models

In the basic research on TBI, free-fall models, controlled cortical impact models, and fluid percussion injury models are frequently utilized (128–130). In initial investigations, the research team utilized an enhanced Feeney free-fall injury model to develop a rat model of TBI (2, 3, 131).

In the field of biomaterials research, it has been established that models of TBI can be effectively created through the excision of tissue. The remediation of brain tissue defects caused by injury has become a significant focus and challenge within the research community. According to the literature, the administration of C1A1 porous hydrogels into murine models with brain tissue defects has shown that these hydrogels can function effectively as scaffolds for brain parenchymal defects. This approach facilitates cellular infiltration and vascularization, thereby enhancing the reconstruction of brain tissue (132). In a study investigating the repair mechanisms of TBI, rat brain parenchyma was surgically excised, and a hydrogel characterized by robust adhesive and haemostatic properties was administered into the resulting defect site. The findings indicated that the hydrogel not only exhibited antibacterial and haemostatic effects but also facilitated neural repair and expedited wound healing (81). After excising a 5 mm × 3 mm × 3 mm section of brain tissue in rats and subsequently injecting hyaluronic acid-KLT (VEGF mimetic peptide, sequence is KLTWQELYQLKYKGI) hydrogel (HA-KLT) into the resulting defect cavity, it was observed that the hydrogel facilitates angiogenesis and tissue regeneration (133). In studies involving mouse brain injuries, the excision of damaged brain tissue followed by the administration of 10 μL of conductive microporous hydrogel (CMH) into the injury site has demonstrated that this hydrogel possesses anti-inflammatory and pro-angiogenic properties. These effects contribute to the restoration of brain functional connectivity and indicate its potential for neurotherapeutic applications (134). In a C57 mouse brain tissue excision model, *in situ* injected hyaluronic acid/gelatin/salvianolic acid B/vascular endothelial growth factor hydrogel (HA/Gel/SAB/VEGF) effectively filled the tissue defect cavity, enhancing angiogenesis and brain tissue regeneration (135) (see Table 3).

**Table 3:** Research on injectable hydrogels in different brain injury models.

Time	Animal	Size of tissue excision	Injectable hydrogel	Function
2024	Mice	1 mm × 1 mm × 1 mm	HA/Gel/SAB/VEGF hydrogel	Promotes angiogenesis and brain tissue regeneration (135)
2024	SD	3 mm × 3 mm × 3 mm	COCS hydrogel	Haemostasis, antibacterial, self-healing, promoting vascular regeneration, enhancing learning and memory (81)
2023	Mice	1 mm × 1 mm × 1 mm	C1A1	Promote angiogenesis and tissue regeneration (132)
2022	Mice	10 uL	CMH	Alleviate inflammation, promote angiogenesis and neuronal survival, and facilitate brain tissue regeneration (134)
2019	SD	5 mm × 3 mm × 3 mm	HA-KLT hydrogel	Angiogenesis (133).

In contrast to conventional controlled cortical impact (CCI), fluid percussion injury (FPI), and Feeney free-fall models, brain tissue resection primarily mitigates the compression and displacement of brain parenchyma associated with hydrogel injection into the brain substance, thereby averting the subsequent development of brain herniation. The excision of damaged brain tissue diminishes the release of inflammatory mediators, free radicals, and excitotoxic substances from the injured regions, which are known to induce neuronal apoptosis and glial scar formation. By removing necrotic tissue, this intervention disrupts the detrimental cycle. The resultant cavities provide a physical space for hydrogel filling, thereby preventing local tissue collapse. This approach stabilizes structural integrity while facilitating tissue remodeling and regeneration (136). This serves as a reference animal model for the investigation of biomaterials, with a specific focus on injectable hydrogels, in the context of localized drug delivery subsequent to TBI.

## 6 Conclusion and future perspectives

The treatment of TBI through conventional pharmacotherapy remains problematic due to challenges such as the BBB and off-target toxicity. These limitations have catalyzed the development of injectable hydrogels, which facilitate localized drug delivery, provide a supportive three-dimensional microenvironment, and allow for minimally invasive administration. Preclinical studies have shown that injectable hydrogels can promote neural repair and functional recovery in animal models of TBI, underscoring their potential in managing secondary injury. To guide clinical operation, an ideal injectable hydrogel for TBI should possess key physical properties: rapid gelation, shear-thinning injectability, tissue-matching modulus (100–1,000 Pa, soft yet supportive), hemostatic adhesion, and controlled degradation (matching neural regeneration). These features directly address surgeons’ intraoperative and postoperative needs.

Future research should focus on three strategic directions: (1) intelligent, stimuli-responsive hydrogels for pathology-triggered drug delivery; (2) brain-specific, extracellular matrix-mimetic hydrogels to enhance cellular integration; (3) multifunctional composite systems integrating hydrogels with complementary therapeutic modalities. Collectively, these strategies aim to establish next-generation intelligent biomaterials for precise repair of the injured brain.
