# Supplementary material for: Dynamic management of traumatic brain injury in rat: injectable hydrogels and future directions
Source: Front Neurol. 2026 Apr 30;17:1824220. doi: 10.3389/fneur.2026.1824220 (PMC13171402; doi:10.3389/fneur.2026.1824220)
Supplement: Supplementary file 1 [file Supplementary_file_1.docx]

**Appendix Table 1. List of Abbreviations and Their Full Terms.**

| **Abbreviations** | **Full terms** |
| --- | --- |
| TBI | Traumatic brain injury |
| BBB | Blood-brain barrier |
| ROS | Reactive oxygen species |
| NMDA | N-methyl-D-aspartate |
| AMPA | α-amino-3-hydroxy-5-methyl-4-isoxazolepropionic acid |
| NO | Nitric oxide |
| NOS | Nitric oxide synthase |
| TNF-α | Tumor Necrosis Factor-alpha |
| IL-1β | Interleukin-1 beta |
| IL-6 | Interleukin-6 |
| NSAIDs | Non-steroidal anti-inflammatory drugs |
| DEX | Dexamethasone |
| T1AM | Triiodothyronine amine |
| ATP | Adenosine triphosphate |
| CCI | Controlled cortical impact |
| FPI | Fluid percussion injury |
| CMH | Conductive microporous hydrogel |
| QCS | quaternised chitosan |
| TA | tannic acid |
| MBs | Gelatin methacryloyl microbeads |
| OSA | Oxidized Sodium Alginate |
