# Supplementary material for: Dynamic management of traumatic brain injury in rat: injectable hydrogels and future directions
Source: Front Neurol. 2026 Apr 30;17:1824220. doi: 10.3389/fneur.2026.1824220 (PMC13171402; doi:10.3389/fneur.2026.1824220)
Supplement: Supplementary file 2 [file Supplementary_file_2.docx]

**Appendix Table 2. List of the chemical composition and full terms of hydrogels.**

| **Abbreviations** | **Full terms of hydrogels** | **Chemical composition** | **Chemical classes** |
| --- | --- | --- | --- |
| HA-PBA/Gel-Dopa | Phenylboronic acid grafted hyaluronic acid/Dopamine grafted gelatin | Phenylboronic acid grafted hyaluronic acid,  dopamine grafted gelatin | Composite hydrogel |
| COCS | Ca²⁺-Crosslinked Oxidized Sodium Alginate (OSA) and Carboxymethyl Chitosan hydrogel | oxidized sodium alginate, carboxymethyl chitosan | Natural polymer hydrogel |
| Bup/PO | bupivacaine-loaded poloxamer/oxidized hyaluronic acid hydrogel | Bupivacaine, aminated poloxamer 407,  oxidized hyaluronic acid | Composite hydrogel |
| HA-PVA-Hep | hyaluronic acid-poly (vinyl alcohol)-heparin hydrogel | Phenylboronic acid and cysteamine modified hyaluronic acid, poly (vinyl alcohol), maleimide functionalized heparin | Composite hydrogel |
| [PPCNW@dGel](mailto:PPCNW@dGel) | polydopamine-mediated poly(3,4-ethylenedioxythiophene)-modified cellulose nanowhisker@dGel hydrogel | Cellulose nano-whiskers, polyethylene glycol diacrylate,  polyethylene glycol diglycidyl ether | Nanocomposite hydrogel |
| Pol/T | poloxamer hydrogel loaded with 3-iodothyronamine | Poloxamer 407, poloxamer 188, 3-iodothyronamine | Composite hydrogel |
| P-RT/2DG | polyethylene glycol-ROS responsive thioketal linker/2-deoxyglucose hydrogel | Polyethylene glycol, thioketal linker, 2-deoxyglucose | Composite hydrogel |
| HA-PBA/PVA/DFO | phenylboronic acid grafted hyaluronic acid/polyvinyl alcohol/deferoxamine hydrogel | Phenylboronic acid grafted hyaluronic acid, polyvinyl alcohol, deferoxamine mesylate | Composite hydrogel |
| lysine/PEGDA | curcumin-loaded lysine/poly (ethylene glycol) diacrylate | Poly (ethylene glycol) diacrylate, lysine, curcumin | Composite hydrogel |
| TM/PC | triglycerol monostearate/poly (propylene sulfide)120 embedded Curcumin Hydrogel | Poly (propylene sulfide) 120, triglycerol monostearate, curcumin | Composite hydrogel |
| GelMA-PPS/PC | gelatin methacryloyl-poly(propylene sulfide)/procyanidins hydroge | Gelatin Methacrylate, poly (propylene sulfide) 60, procyanidins | Composite hydrogel |
| PDA-G-A-H | polydopamine-gentamicin-alendronate-hydrogel | Polydopamine nanoparticles, gentamycin, alendronate acid, pluronic F127, collagen | Nanocomposite Hydrogel |
| PLGA- HA,  HAAC/GelMA | poly(dl-lactide-co-glycolide)-hyaluronic acid； hyaluronic acid adipic dihydrazide/gelatin methacryloyl hydrogel | Poly (dl-lactide-co-glycolide), hyaluronic acid, acrylated hyaluronic acid, gelatin methacryloyl, thiolated heparin | Composite hydrogel |
| AOHA-RA/Lap IH | 4-aminobenzeneboronic acid grafted oxidized hyaluronic acid / rosmarinic acid / laponite injectable hydrogel | 4-aminobenzeneboronic acid grafted oxidized hyaluronic acid, rosmarinic acid, laponite | Nanocomposite Hydrogel |
| [HAMA/COS/MSN@BMP-4](mailto:HAMA/COS/MSN@BMP-4) | methylpropenylated hyaluronic acid/chitosan oligosaccharide/mesoporous silica nanoparticles@bone morphogenetic protein-4 hydrogel | Methylpropenylated hyaluronic acid, chitosan oligosaccharide, mesoporous silica nanoparticles, bone morphogenetic protein-4 | Nanocomposite Hydrogel |
| HA-KLT | hyaluronic acid-KLT | Hyaluronic acid, KLTWQELYQLKYKGI (VEGF-mimetic peptide) | Composite hydrogel |
| HA/Gel/SAB/VEGF | hyaluronic acid/gelatin/salvianolic acid B/vascular endothelial growth factor | Hyaluronic acid, gelatin, salvianolic acid B,  vascular endothelial growth factor | Composite hydrogel |
